# Motivation Classification and Grade Prediction for MOOCs Learners

**DOI:** 10.1155/2016/2174613

**Published:** 2016-01-14

**Authors:** Bin Xu, Dan Yang

**Affiliations:** ^1^Computing Center, Northeastern University, Shenyang 110819, China; ^2^College of Information Science and Engineering, Northeastern University, Shenyang 110819, China

## Abstract

While MOOCs offer educational data on a new scale, many educators find great potential of the big data including detailed activity records of every learner. A learner's behavior such as if a learner will drop out from the course can be predicted. How to provide an effective, economical, and scalable method to detect cheating on tests such as surrogate exam-taker is a challenging problem. In this paper, we present a grade predicting method that uses student activity features to predict whether a learner may get a certification if he/she takes a test. The method consists of two-step classifications: motivation classification (MC) and grade classification (GC). The MC divides all learners into three groups including certification earning, video watching, and course sampling. The GC then predicts a certification earning learner may or may not obtain a certification. Our experiment shows that the proposed method can fit the classification model at a fine scale and it is possible to find a surrogate exam-taker.

## 1. Introduction

Over the past three years, benefiting from innovative cloud computing technologies, Massive Open Online Courses (MOOCs) bring us many top courses which are provided by top academic institutions. Tens of millions of students from all over the world were attracted to join these courses. This also led to changes of higher education [1]. Currently, more and more courses are uploaded to MOOCs platforms such as Coursera and Edx by an increasing number of universities. Some universities choose to join together to provide better and more comprehensive courses, including Harvard, MIT. Besides North America, other regions have also provided MOOCs platform, such as FutureLearn and Iversity in Europe. With the most number of college students, universities in China also began to join existing MOOCs platform or develop a new one.

MOOCs allow anyone in the world to study a course by accessing course resources or watching videos freely, and the great attraction brought together a large number of learners in a short time. In the largest MOOCs platform Coursera, for example, the current total registered users have exceeded 14 million and the number is still increasing every second.

Unlike traditional online courses which just provide curriculum materials for downloading, MOOCs integrate teaching process into learning. For example, instructors will release teaching video according to a planned schedule, and learners should submit their homework by deadline. After finishing final exams, the course will be closed. After that, registers can only watch archived videos or discuss them in course forum but can not submit assignments any more. For those learners who pass final exams, they may receive a certification from course institutions. Compared with the traditional university course, most of these courses are totally free and open for everyone even if he/she is only a middle school student. Grading every learner is a difficult task because of the huge number of learners involved in the courses and many problems in homework or exam cannot be graded by program automatically. Peer assessment is one option to deal with this problem. However, how to guarantee the justice of every assessment or how to find out surrogate exam-takers is essential for all learners.

Fortunately, in addition to providing excellent learning resources, MOOCs platform also saves detailed activity records of massive learners during the learning process. As the saying “no pain, no gain,” the score of one learner is relative to his/her learning engagement. Based on this rule, the score can be predicted by analyzing his/her learning activities. An abnormal assessment can be detected by comparing the predicted score with final score. However, how to deal with the massive data is still a challenge. The learning behaviors of large numbers of learners including clickstream of video watching are fully tracked in the data. The record of one course is even more than 10 GB [2]. This brings higher education into the era of big data. Based on Person-Course Dataset AY2013 which is provided by HarvardX-MITx, the motivation of learners is classified in this paper according to their activity records; then final grade is predicted for those learners trying to earn their certification.

## 2. Related Works

Some researchers [3, 4] thought that MOOCs will play an important role in our future education and even change it. A growing number of investigators began to carry out relative researches on MOOCs recently. These researches focus on two aspects mainly. The first is how to improve the MOOCs platform in personalization or to provide new functions for both learners and instructors. For example, J. J. Williams and B. Williams [5] investigated how varying reminders and resources sent through emails to participants influence their use of course components like forums and their overall outcomes. Diez et al. [6] presented a matrix factorization approach able to learn from the order of the subset of exams evaluated by each grader. Shah et al. [7] explored an ordinal approach to peer evaluation based on pairwise comparisons to improve robustness. The second aspect is to explore cognitive rules of learner by analyzing learning behavior and therefore to predict their following behavior such as whether he/she will quit the course. Yang et al. [8] developed a survival model that allows us to measure the influence of factors extracted from learning behavior data on student dropout rate. Ramesh et al. [9] used probabilistic soft logic (PSL) to model student engagement by capturing domain knowledge about student interactions and performance. Balakrishnan [10] used a combination of students' Week 1 assignment performance and social interaction within the MOOCs to predict their final performance in the course. Kizilcec et al. [11] presented a simple, scalable, and informative classification method that identifies a small number of longitudinal engagement trajectories in MOOCs. This paper focuses on predicting the probability of students to earn certificates for completion of the MOOCs based on classification of learners. Learner classification can be fulfilled by different criteria. Researchers from Stanford [12] divided learners into five categories by analyzing learning activities such as viewing a lecture and handing in an assignment for credit: Viewers, Solvers, All-rounders, Collectors, and Bystanders. Researchers from MIT [13] divided learners into four types based on whether or not they participated in the class forum or helped edit the class wiki pages: passive collaborator, wiki contributor, forum contributor, and fully collaborative. For more in-depth analysis of learning behavior, behavioral model is established. Sinha et al. [14] constructed a detailed encoding of students' clicks in the following 8 categories: Play, Pause, SeekFw, SeekBw, ScrollFw, ScrollBw, RatechangeFast, and RatechangeSlow by analyzing clickstream data and then seven different behavior models including Rewatch, Skipping, Fast Watching, Slow Watching, Clear Concept, Checkback, and Playrate Transition are identified. Information Processing Index (IPI) is then calculated by assigning different weights to different behavior models to measure engagement and interest for every learner. Hidden Markov Model (HMM) [10] or logistic regression model [13] was introduced to predict learner's dropout behavior. 27 different behavioral characteristics of learners were collected and fed to a binary classifier [13]. Furthermore, the parameters are automatically optimized based on machine learning services cloud framework [15]. To find out what the confusion for one learner is during learning process, Agrawal et al. [16] adopted crowdsourcing to label every post in the course forum from six aspects including the following: “Is a post a question?” “Does a post answer a question?” “What is the confusion level for a post?” “What is the urgency of a post?” A feature space was constructed by combining these labels with post content and metadata. Then the confusion was measured through a compound classifier. The authors also pointed out that the behavior pattern modeling and learning performance evaluation are much more complicated than expected and still have a long way to go.

## 3. Dataset Description

This paper aims to find out the difference between different learners in activity features by analyzing learning activities data from MOOCs. The Person-Course Dataset AY2013 contains 16 courses including course information such as course ID, open date, launch date, and learner activities such as video play activity and course forum activity. Six courses are excluded from 16 courses because of insufficient activity data.  Table 1 shows the basic information of 10 selected courses.

**Figure 1:**
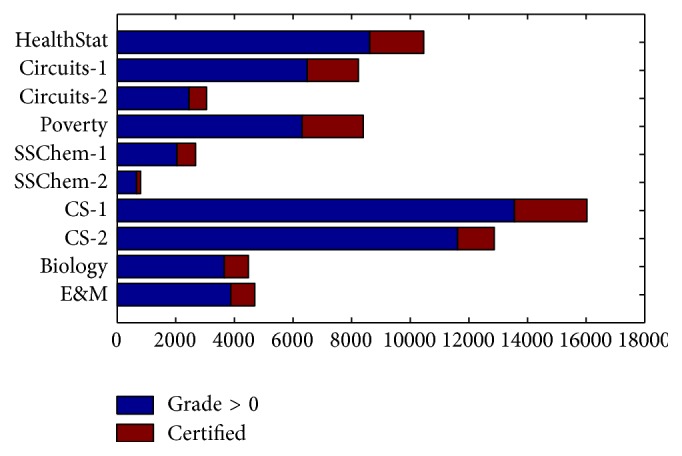
The number of exam-takers and who really get certified.

Although the number of enrolled learners is enormous, only a small partial of them completed the whole course from the beginning to the end and took the final exam. As reported, the average complete rate is less than 10% [17]. Furthermore, the number of learners who took final exams and obtained certification is even lower.  Figure 1 shows the learners who took exams and obtained a certification. We consider that all those grades are greater than zero as final exam-taker. The average certificate rate of 10 courses is 23%. Even though it is a low rate, the number of exam-takers for every course still may be greater than 10 thousand and therefore the grading task is still a burdensome work. A predicted grade is helpful in avoiding grading mistake or in detecting cheating on the exams.

## 4. Learning Types

Due to the great diversity of learners in age, education background, region, motivation, and learning habits, grade predicting is a big challenge in MOOCs. 13 different kinds of motivation are listed in paper [18]. Online survey is a good option to recognize learners' motivation, but most of them may not respond to an online survey [19]. According to psychology, particular behavior of someone may reflect his/her evaluation for a positive excitation. Therefore, learning activities may reflect a learner's motivation. The detailed records of learning activities in MOOCs platforms give us a chance to analyze a learner's motivation. Based on this theory, we divided all learners into three categories.

### 4.1. Certification Earning

Aiming at earning a certification, these learners always complete the course to the end and take final exams. Some learners do not take final exams even if they complete the course. This explains that their target is not a certification.

### 4.2. Video Watching

Aiming at acquisition of course knowledge, these learners always have high video playing activities. They may select some of videos which they are interested in to study. If possible, they may submit some homework. Some of them take the course as supplement to their college courses. Some of them will take final exams if possible.

### 4.3. Course Sampling

These learners just come to check the content of the course or make sure if the course meets their need. Some of them may watch one or two videos but they may not submit any homework.

**Figure 2:**
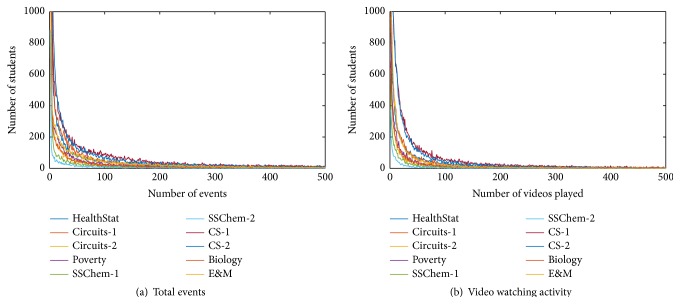
The number of learners with same activity.

Video watching is the most important way of learning in MOOCs. Therefore, we focus on video playback activity of learners. In given dataset, every activity is collected as an event. When a learner logs in to a course or watches a video or posts a message in course forum, this will generate a new event. For comparison, Figure 2 shows the number of learners with the same video watching activity and the same total events.

Both total event activity and video watching activity show consistent decline in the number of learners. We adopt an exponential function *f*(*x*) = *a* × *e* ^−*bx*^ + *c* × *e* ^−*dx*^ to fit these curves where the first term represents the video playing activities and the second term stands for any other activities except video watching.  Table 2 shows the function parameters of every course for total event activity and corresponding evaluation measurement where root mean squared error (RMSE) is known as the fit standard error and the standard error of the regression and *R*-square measures how successful the fit is in explaining the variation of the data.

For all 10 courses, *R*-square measurements are all greater than 0.986. The curve fitting result shows that fitting curve and original curve have a high correlation. Although the difference in parameters *a* and *c* for different courses is great (standard deviation of parameter *a* is equal to 5617 and standard deviation of parameter *c* is equal to 192), the difference for parameters *b* and *d* is really small (standard deviation of parameter *b* equals 0.1056 and standard deviation of parameter *d* equals 0.0044). The great difference in parameters *a* and *c* is caused by the great different in the number of enrollment learners for different course.

**Figure 3:**
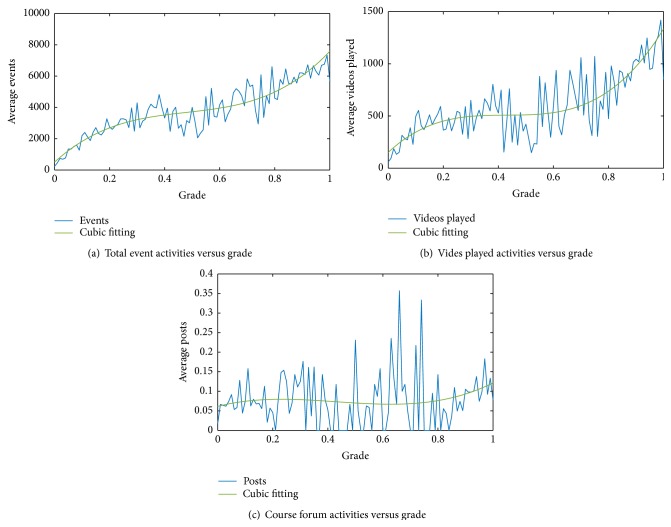
Learner's activities versus grade of course HealthStat.

Figure 3 shows the relationship between learner's activities and grades of the course HealthStat. The grade is a value between 0 and 1 which means the score of the exam of all learners taking the exam. Both total event activities and video playing activities show consistent relationship with grade. The grade can be split into three stages. The first stage is grade less than 0.3. The activity was significantly less than normal during this stage with average video watching activities less than 500. Most of the learners in this stage failed to submit their homework or quit learning after few weeks. Course sampling is the main motivation for these learners. The second stage is grade between 0.3 and 0.7. The activities of learners in this stage fluctuate dramatically. Some of them have higher video playing activities while some more are active in the course forum. Video watching or certification earning could be motivation for these learners. The third stage is grade higher than 0.7. The fluctuations of activities tend to be small while the grade is rising rapidly. Certification earning is the main motivation for these learners. The fitting curves also proved the assumption of the grade of one learner being relative to his/her learning engagement; that is, if a learner engages more time in learning process, he/she will more likely get higher score. However, post activities in the course forum are steady for all grades and do not show clear correlation with grades. This explains that video watching is more important than forum post activities to gain high grades.

**Figure 4:**
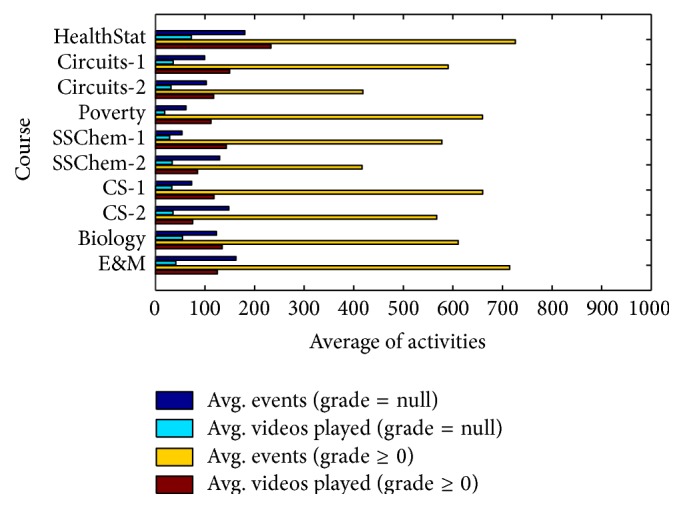
Average activities of exam-taker and non-exam-taker.

Figure 4 shows the average total event activities and average video watching activities of two group learners: exam-takers and non-exam-takers. The difference between these two groups is apparently huge especially in total event activities. The average event activities of exam-takers are three times more than non-exam-takers at least. If a learner wants to take final exams, he/she will spend more time on this course.

Based on the above analysis, learners can be divided into different categories according to their activities. Learners in category A (certification earning) have highest activities while learners in category C (course sampling) have lowest activities. An activity index value was proposed to measure the engagement of a learner. According to above statistics, if a learner spend more time (days) in one course or with higher event activities especially video playing activities, he/she should obtain a higher activity value, while if a learner enrolled on too many courses, the engagement in one course will be less. After repeated attempts, the activity index *P*_*a*_ is finally defined as follows:

(1) $$\begin{array}{c} P_{a}=k_{1}\frac{f_{d}\left(d\right)\sqrt{f_{e}\left(e\right)f_{v}\left(v\right)}}{f_{c}\left(c\right)}+k_{2}f_{t}\left(\;t\;\right), \end{array}$$

where *d*, *e*, *v*, *c*, and *t* are activity features for a learner. Each of them is defined, respectively, as follows: *d* is the feature for learners' active days, *e* is the feature for total activities, and *v* is the feature for video playback activities. These features are extracted as follows.

*d* = *d*_*u*_/*d*_*l*_ is feature for learners active days, wherein *d*_*u*_ is the cumulative active days of a learner and *d*_*l*_ is days span of this learner. This feature describes the frequency of a learner to take this course. For example, if a learner watches video once a week, the *d* value is close to 1/7.

*e* = *e*_*u*_/*e*_*m*_ is feature for learners total event activity, wherein *e*_*u*_ is total event activities of a learner and *e*_*m*_ is the maximum event activities in the course. This feature describes the level of activity of learners in a course.

*v* = *v*_*u*_/*v*_*a*_^2^ is feature for learners video watching activity, wherein *v*_*u*_ is video watching activities of a learner and *v*_*a*_ is the average video watching activities of all learners in the course. This feature describes the level of video watching activity in a course.

*c* = *c*_*u*_/*K*_*c*_ is feature for learners course enrollment activity, wherein *c*_*u*_ is total enrolled courses of a learner and *K*_*c*_ is the total courses in dataset.

*t* is feature for learners exam taken activity. It is a binary value where 1 is for exam-taker and 0 is for non-exam-taker.

*f*_*d*_, *f*_*e*_, *f*_*v*_, *f*_*c*_, *f*_*t*_ are function corresponding to the above five features. Based on experiment, *f*_*d*_, *f*_*c*_ are sigmoid function, *f*_*e*_, *f*_*v*_ are power function, and *f*_*t*_ is linear function. *k*_1_ and *k*_2_ are two constant coefficients.

**Figure 5:**
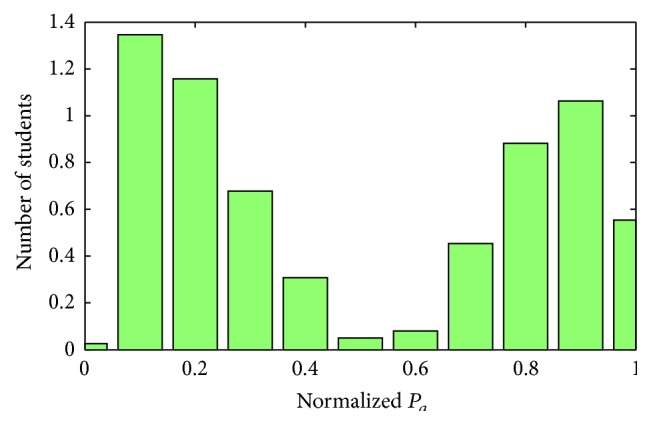
Number of students of different activity index (Course: CS-1).

Figure 5 shows the number of learners of course CS-1 in terms of normalized *P*_*a*_ value.

**Figure 6:**
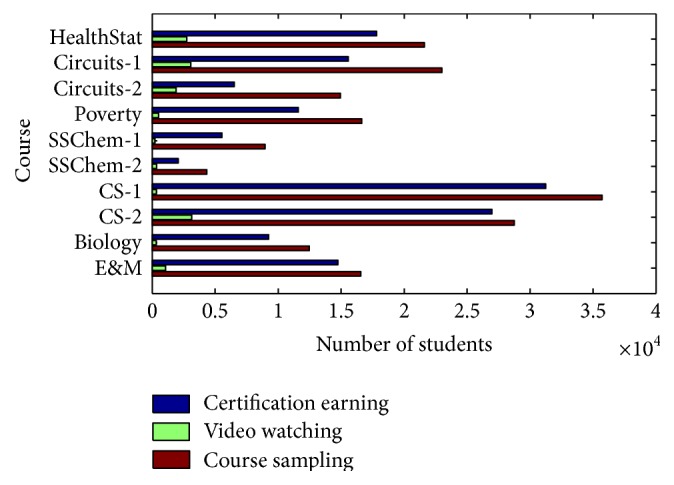
Comparison of the number of three different categories.

Eventually, all learners in a course are divided into three categories. For example, in course CS-1 as shown in Figure 5, those learners with *P*_*a*_ value less than 0.5 are grouped as course sampling and learners with *P*_*a*_ value greater than 0.6 are grouped as certification earning.  Figure 6 shows the number of learners in three categories of 10 courses. Course sampling category is the largest group for all 10 courses. This is also a good explanation for low complete rate in MOOCs because many of them are course sampling learners. These learners tend to drop out after a few weeks. Video watching category is the smallest group for all 10 courses. If someone watches many videos, he/she may probably be incapable of gaining a certification even if he/she does not intend to gain one at the beginning of the course because certification is an important additional option for learners. That is, some video watching learners will transform to certification earning learners with the course progress.

## 5. Grade Prediction

For MOOCs, we hope to predict the final grade for learners. This may be helpful for saving instructor's time or finding surrogate exam-takers. Depending on the activities of learners, their final grade can be predicted before final exams. According to previous classifications, learners with different motivation will lead to different activity features. For example, the video playing activities of a certification earning learner always occurs at a fixed interval while a video watching learner may play videos at random time and not last for the whole course period. Therefore, different groups should be predicted with different models. Group C (*course sampling*) was excluded because most of them would not take final exams. And certification earning learners are much more than video watching learners. Therefore, we try to predict any certification earning learner if he/she will gain a certification. All activities including videos played (*x*_1_), posts in course forum (*x*_2_), active days (*x*_3_), days between first event and course opening date (*x*_4_), days between last event and course closing date (*x*_5_), and enrolled courses (*x*_6_) are resampled for every certification earning learner. All these learners are randomly divided into training set and test set by ratio 3 : 2. The training set is used for training parameters and test is used set for prediction performance evaluation. Therefore, all learners can be expressed as

(2) $$\begin{array}{c} x=\left\{\;x_{1},x_{2},x_{3},x_{4},x_{5},x_{6}\;\right\}. \end{array}$$

Support Vector Machine (SVM) is a wide accepted supervised learning model with associated learning algorithms that analyze data and recognize patterns, used for classification and regression analysis. A SVM-based model is proposed to classify all certification learners into two classes: a learner may or may not obtain a certification. The prediction problem can be represented as follows.

For *m* learners, there is a feature matrix **S** ∈ *R* ^*m*×*n*^ and prediction result **Y** = *f*(**S**) where **Y** is *m*-dimensional binary vector whose value comes from {0,1}. We take Gaussian RBF kernels of the form *K*(**x**_1_, **x**_2_) = *e* ^−‖**x**_1_ − **x**_2_‖/2*σ*^2^^and consider the decision function evaluated on the support vector **s**_*j*_:

(3) $$\begin{array}{c} f\left(\;s_{j}\;\right)=\sum_{i}\alpha_{i}y_{i}e^{-{\left\|s_{i}-s_{j}\right\|}^{2}/2\sigma^{2}}+b. \end{array}$$

**Figure 7:**
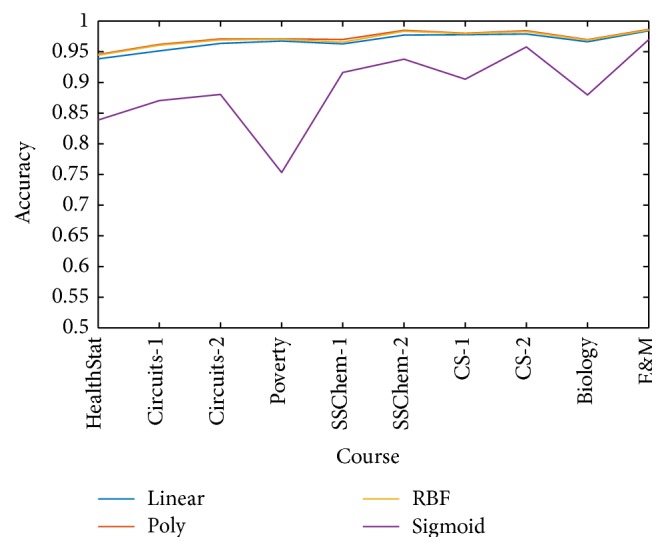
Prediction accuracy of different SVM kernels.

For comparison, different SVM kernels are selected for prediction including linear kernel, poly kernel, and sigmoid kernel. Accuracy was calculated from predicted grade and true grade to measure performance of grade prediction:

(4) $$\begin{array}{c} \text{Accuracy}=\frac{TP+TN}{TP+TN+FP+FN}, \end{array}$$

where TP, FP, TN, and FN are number of true positives, false positives, true negatives, and false negatives, respectively.  Figure 7 shows the prediction accuracy of different kernels.

## 6. Conclusion

Based on their activities in MOOCs, learners are classified into different groups by their motivation. After that, grade prediction is applied to those certification earning learners. Prediction accuracy is improved due to the fact that the parameters of classification model can be tuned in a finer scale to fit more learners. However, if we want to predict specific grade value but not only if a learner will earn a certification or not, the classifier should be resigned to fit more targets classification application such as predicting learners into several levels, for example, to predict a leaner's grade as five levels such as A, B, C, D, and E. On the other hand, learners may join a course with the motivation to persist for some or the entire course, but various factors, such as attrition or lack of satisfaction, can lead them to disengage or totally drop out. How to deal with the motivation transition is also a problem to be solved in future.

## Figures and Tables

**Table 1:** Courses information.

Course	Semester	Registration open date	Launch date	Wrap date
HealthStat	Fall 2012	2012/7/24	2012/10/15	2013/1/30
Circuits-1	Fall 2012	2012/7/24	2012/9/5	2012/12/25
Circuits-2	Spring 2013	2012/12/20	2013/3/3	2013/7/1
Poverty	Spring 2013	2012/12/19	2013/2/12	2013/5/21
SSChem-1	Fall 2012	2012/7/24	2012/10/9	2013/1/15
SSChem-2	Spring 2013	2012/12/20	2013/2/5	2013/6/21
CS-1	Fall 2012	2012/7/24	2012/9/26	2013/1/15
CS-2	Spring 2013	2012/12/19	2013/2/4	2013/6/4
Biology	Spring 2013	2013/1/30	2013/3/5	2013/6/6
E&M	Spring 2013	2013/1/17	2013/2/18	2013/6/18

**Table 2:** Curve fitting parameters for total event activity.

Course	*a*	*b*	*c*	*d*	RMSE	*R*-square
HealthStat	11650	0.7143	400	0.028	6.976	0.9959
Circuits-1	8109	0.5785	402	0.024	6.821	0.9956
Circuits-2	6441	0.6945	215	0.029	5.115	0.9966
Poverty	7396	0.8315	287	0.029	4.306	0.9963
SSChem-1	1864	0.7852	58	0.023	2.747	0.9981
SSChem-2	2098	0.9321	62	0.024	2.829	0.9925
CS-1	21170	0.7453	447	0.018	10.52	0.9959
CS-2	8144	0.6076	701	0.031	10.9	0.9892
Biology	4932	0.8031	212	0.028	4.514	0.9931
E&M	4257	0.7996	263	0.019	5.922	0.9860
